# Post-Acute COVID-19 Joint Pain and New Onset of Rheumatic Musculoskeletal Diseases: A Systematic Review

**DOI:** 10.3390/diagnostics13111850

**Published:** 2023-05-25

**Authors:** Jacopo Ciaffi, Elena Vanni, Luana Mancarella, Veronica Brusi, Lucia Lisi, Federica Pignatti, Susanna Naldi, Elisa Assirelli, Simona Neri, Massimo Reta, Cesare Faldini, Francesco Ursini

**Affiliations:** 1 Medicine & Rheumatology Unit, IRCCS Istituto Ortopedico Rizzoli (IOR), 40136 Bologna, Italy; elena.vanni97@gmail.com (E.V.); luana.mancarella@ior.it (L.M.); veronica.brusi@ior.it (V.B.); lucia.lisi@ior.it (L.L.); federica.pignatti@ior.it (F.P.); susanna.naldi@ior.it (S.N.); elisa.assirelli@ior.it (E.A.); simona.neri@ior.it (S.N.); francesco.ursini2@unibo.it (F.U.); 2 UO Interaziendale Medicina Interna ad Indirizzo Reumatologico (SC) AUSL BO—IRCCS AOU BO, 40133 Bologna, Italy; massimo.reta@ausl.bologna.it; 3 Department of Biomedical and Neuromotor Sciences (DIBINEM), University of Bologna, 40126 Bologna, Italy; cesare.faldini@ior.it; 4 1st Orthopedic and Traumatology Clinic, IRCCS Istituto Ortopedico Rizzoli (IOR), 40136 Bologna, Italy

**Keywords:** COVID, post-COVID, musculoskeletal, pain, arthralgia, arthritis, autoantibodies, fibromyalgia

## Abstract

As the number of reports of post-acute COVID-19 musculoskeletal manifestations is rapidly rising, it is important to summarize the current available literature in order to shed light on this new and not fully understood phenomenon. Therefore, we conducted a systematic review to provide an updated picture of post-acute COVID-19 musculoskeletal manifestations of potential rheumatological interest, with a particular focus on joint pain, new onset of rheumatic musculoskeletal diseases and presence of autoantibodies related to inflammatory arthritis such as rheumatoid factor and anti-citrullinated protein antibodies. We included 54 original papers in our systematic review. The prevalence of arthralgia was found to range from 2% to 65% within a time frame varying from 4 weeks to 12 months after acute SARS-CoV-2 infection. Inflammatory arthritis was also reported with various clinical phenotypes such as symmetrical polyarthritis with RA-like pattern similar to other prototypical viral arthritis, polymyalgia-like symptoms, or acute monoarthritis and oligoarthritis of large joints resembling reactive arthritis. Moreover, high figures of post-COVID-19 patients fulfilling the classification criteria for fibromyalgia were found, ranging from 31% to 40%. Finally, the available literature about prevalence of rheumatoid factor and anti-citrullinated protein antibodies was largely inconsistent. In conclusion, manifestations of rheumatological interest such as joint pain, new-onset inflammatory arthritis and fibromyalgia are frequently reported after COVID-19, highlighting the potential role of SARS-CoV-2 as a trigger for the development of autoimmune conditions and rheumatic musculoskeletal diseases.

## 1. Introduction

Viral infections are a well-recognized cause of joint pain and of inflammatory arthritis [1]. Several viruses, such as parvovirus B19, hepatitis viruses, enteroviruses, rubella, flaviviruses, chikungunya virus and HIV, have been associated with different forms of arthritis, ranging from transient self-limiting episodes of joint inflammation to chronic disabling conditions [1]. Joint pain, muscle pain and fatigue are also present in a high proportion of patients with COVID-19, the disease caused by the severe acute respiratory syndrome coronavirus 2 (SARS-CoV-2), both as initial presentation or during the course of the illness [2]. Although patients with COVID-19 can recover completely, a considerable number of individuals experience persistent symptoms after the acute phase of the infection and may develop a broad spectrum of sequelae [3]. Beyond pulmonary complications, frequent COVID-19 sequelae include cardiovascular, neurological or hematological manifestations and other less common long-term effects [4]. Angiotensin-converting enzyme 2, the host entry receptor of SARS-CoV-2 in human cells [5], is expressed in the musculoskeletal system, in particular in skeletal muscle and synovial tissue cells [6,7]. Not surprisingly, musculoskeletal symptoms are reported in a significant proportion of post-COVID-19 patients [8]. A growing number of articles described new-onset rheumatic musculoskeletal diseases developing in close temporal association with COVID-19, including rheumatoid arthritis (RA) [9], polymyalgia rheumatica (PMR) [10], reactive arthritis [11], axial spondyloarthritis [12], polyenthesitis [13] and connective tissue diseases [14,15,16]. Furthermore, current evidence suggests not only that various autoantibodies can be found in the sera of individuals recovered from SARS-CoV-2 infection [17,18,19], but also that the long-lasting presence of autoantibodies might be associated with persisting symptoms and residual inflammation [20]. From the rheumatologist’s perspective, the impact of musculoskeletal manifestations in post-COVID-19 patients is a rapidly evolving field. Since data about the long-term effects of SARS-CoV-2 infection are continuously rising, it is important to summarize the available literature. Therefore, the aim of the present systematic review is to provide a more granular picture of post-acute COVID-19 musculoskeletal manifestations of rheumatological interest, with particular attention to: (1) joint pain; (2) new onset of rheumatic musculoskeletal diseases including RA, spondyloarthritis, PMR, gout, myositis and fibromyalgia; (3) presence of autoantibodies related to inflammatory arthritis such as rheumatoid factor (RF) and anti-citrullinated protein antibodies (ACPA). 

## 2. Materials and Methods

### 2.1. Literature Search

MedLine (through PubMed) and Web of Science (WOS) databases were searched up to October 1, 2022. The string used to perform the search in MedLine was (“COVID-19” OR “covid*” OR “2019 novel coronavirus*” OR “2019 ncov*” OR “sars cov 2*” OR “coronavirus disease*” OR “severe acute respiratory syndrome coronavirus 2” OR “wuhan coronavirus*”) AND (“Joint Diseases” [MeSH Terms] OR “arthralgia” OR “Arthralgia” [MeSH Terms] OR “musculoskeletal” OR “tenosynovitis” OR “synovitis” OR “enthesitis” OR “dactylitis” OR “autoantibodies” [MeSH Terms] OR “rheumatoid factor” OR “anticitrullinated peptide antibodies” OR “arthritis” [MeSH Terms] OR “arthri*” OR “polyarthri*” OR “spondylarthropathies” [MeSH Terms] OR “spondyloarthro*” OR “spondylarthro*” OR “myositis” [MeSH Terms] OR “myosit*” OR “polymyositis” OR “dermatomyositis” OR “inflammatory muscle*” OR “inflammatory myopath*” OR “idiopathic inflammatory myo*” OR “still s disease, adult onset” [MeSH Terms] OR “adult onset still*” OR “adult onset still*” OR “fibromy*” OR “fibrositi*” OR “secondary fibromy*” OR “gout” OR “polymyalgia rheumatica”). 

The string used to perform the search in WOS was (“COVID-19" OR “covid*" OR “2019 novel coronavirus*" OR “2019 ncov*” OR “sars cov 2” OR “coronavirus disease*” OR “severe acute respiratory syndrome coronavirus 2” OR “wuhan coronavirus*”) AND (“Joint Diseases” OR “arthralgia” OR “musculoskeletal” OR “tenosynovitis” OR “synovitis” OR “enthesitis” OR “dactylitis” OR “autoantibodies” OR “rheumatoid factor” OR “anticitrullinated peptide antibodies” OR “arthri*” OR “polyarthri*” OR “Ankylosing Spondyloarthritis” OR “spondyloarthro*” OR “spondylarthro*” OR “myosit*” OR “polymyositis” OR “dermatomyositis” OR “inflammatory muscle*” OR “inflammatory myopath*” OR “idiopathic inflammatory myo*” OR “adult onset still*” OR “adult onset still*” OR “fibromy*” OR “fibrositi*” OR “secondary fibromy*” OR “gout” OR “polymyalgia rheumatica”).

The search strategy was designed by one reviewer (EV) and a supervision was granted by a senior investigator (FU). The final manuscript was prepared following the Preferred Reporting Items for Systematic Review and Meta-Analysis (PRISMA) guidelines [http://www.prisma-statement.org/ (accessed on 24 April 2023)].

### 2.2. Eligibility Criteria and Study Selection

Studies were selected if they: (1) were published in the English language; (2) were full-text original articles published in international, peer-reviewed journals; (3) were non-randomized studies or case series detailing 5 or more cases. For the purpose of this review, we defined post-acute COVID-19 as persistent or delayed-onset joint pain or new onset of inflammatory rheumatic musculoskeletal conditions at least 4 weeks after SARS-CoV-2 infection. Articles in which the timing of assessment from SARS-CoV-2 infection was not clearly stated or could not be derived from the available information were excluded, as well as conference proceedings and non-original publications. Moreover, for the evaluation of joint pain, only studies including more than 100 patients were considered eligible. The population, intervention, comparator, outcome (PICO) framework [21] was used to formulate the research question. Studies meeting the following criteria were included in the review:

- Population: patients with a previous confirmed diagnosis of SARS-CoV-2 infection;
- Intervention: assessment of presence of symptoms of rheumatological interest following COVID-19 infection;
- Comparison: no comparison was considered necessary;
- Outcome: prevalence of arthralgia or of autoantibody of rheumatological interest or of an established diagnosis of rheumatic musculoskeletal diseases.

**Figure 1:**
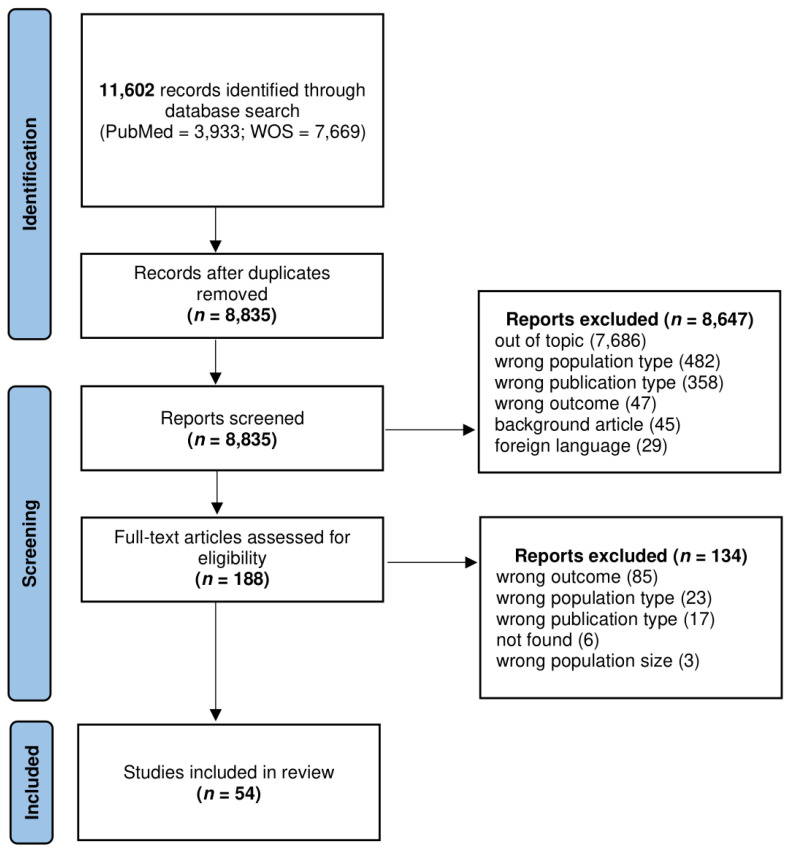
Prisma 2020 flow diagram [24].

A flow diagram of the study selection process is shown in Figure 1. Study identification and data extraction.

After the removal of duplicates, title and abstract screening was performed by two authors (EV and JC) independently. Full-text reading of potentially eligible articles was then performed. References and related citations were also examined to identify additional pertinent papers. Each selected article was summarized and the following information was recorded: first author; year of publication; country; study design; sample size; prevalence of musculoskeletal manifestation of potential rheumatological interest or main findings. The Newcastle–Ottawa scale [22] and the Joanna Briggs Institute critical appraisal tools [23] were used to ensure the quality of non-randomized studies and case series, respectively.

## 3. Results

The search strategy identified 3933 articles from PubMed and 7669 from WOS. After removal of duplicates, 8835 unique articles were selected for title and abstract screening and 188 were considered potentially relevant for full-text evaluation. The full article review identified 54 studies that proceeded to data extraction and analysis. The geographical origin of the included studies was heterogeneous, with a contribution of 23 publications from European centers, 19 studies conducted by Asian teams, 2 undertaken in North America, 5 in South or Central America and 4 in Africa. One study included patients from the United Kingdom, United States and other countries. Characteristics of the selected studies are shown in Table 1. Of the 47 articles evaluated through the Newcastle–Ottawa scale, 18 were rated of good quality, 24 of fair quality and 5 of poor quality (Table 2). Finally, seven case series were deemed eligible after the application of Joanna Briggs critical appraisal tools. 

### 3.1. Joint Pain in Post-COVID-19 Patients

We included 39 studies describing the prevalence of joint pain in post-acute COVID-19 patients. Of these, 7 studies, accounting for a total of 460,716 post-COVID cases, reported a prevalence lower than 10% [36,37,44,59,62,72,77]. The largest was a population-based study involving 456,002 individuals from the United Kingdom at least 4 weeks after COVID-19, where arthralgia was present in 2% to 3% of the patients [72]. In 10 other studies, including 9756 patients overall, a prevalence of joint pain ranging from 10% to 19% was described [35,38,40,41,45,46,51,58,69,73], while 11 studies assessing post-COVID-19 symptoms in 28,303 patients showed higher figures, between 20% and 29% [28,34,42,43,47,49,64,66,71,75,76]. Most of these patients were contributed by Wang et al. [71]. Extracting data from the electronic health records of 26,117 post-COVID-19 individuals, the authors found that arthralgia was one of the most common symptoms in the cohort, having a frequency of 21%. Additionally, 4 studies evaluating data from 7058 patients reported a prevalence between 30% and 39% [31,48,55,63]. Estimates of 40% or above were described in 7 studies on 1748 post-COVID-19 individuals [26,29,33,39,53,57,65]. Finally, data from the U.S. Department of Veterans Affairs electronic health databases showed that, in 73,435 non-hospitalized patients with COVID-19, after a median follow-up of 126 days, there was an excess burden of joint pain (hazard ratio of 5.16, 95% CI 3.18–7.01) compared to almost 5-million users of the Veterans Health Administration who did not have COVID-19 and were not hospitalized [27].

### 3.2. Inflammatory Arthritis in Post-COVID-19 Patients

Inflammatory arthritis in post-COVID-19 patients was reported in eight studies. In a case series of 23 patients with a median of 33 days from COVID-19 diagnosis, Ursini et al. described 15 patients with asymmetric monoarthritis or oligoarthritis, 3 with manifestations suggestive of PMR, 2 with RA-like polyarthritis, 2 with enthesitis and 1 with predominantly axial involvement [67]. Analyzing data from various Dutch rheumatology clinics, Derksen et al. identified 5 patients who developed RA-like polyarthritis after a mean of 7 weeks from infection. Three patients had a clinical presentation compatible with regular patients with new-onset RA and four fulfilled the classification criteria for RA [9]. Similarly, Mukarram et al. reported 5 cases of patients presenting on average 8 weeks after COVID-19 with polyarticular symmetrical inflammatory arthritis involving the small joints of the hands and the wrists, resembling the clinical presentation of RA [56]. Vogler et al. described 4 patients with newly diagnosed arthritis timely related to COVID-19 developing 4–16 weeks after COVID-19. An amount of 1 patient had a polyarthritis, 2 had an oligoarthritis of the upper and lower limb, and in 1 case, a late-onset RA was diagnosed [70]. Gulzar et al. described 5 patients developing arthritis in the 6 months after COVID-19 [42]. Two were patients with arthritis of the ankle joint and one of them had experienced a first gout episode identified by synovial aspirate. Two other patients had a monoarthritis, respectively, of a wrist and of a knee, while one patient with symmetrical arthritis of the small joints of both hands was eventually diagnosed with RA. After 1 year of follow-up on 543 post-COVID-19 patients, Maestre-Muniz found that 13 (2%) had developed a new inflammatory arthritis, but no additional detail was provided [54]. Finally, evaluating a cohort of 100 post-COVID-19 patients and excluding those with pre-existing autoimmune conditions or rheumatic diseases, Taha et al. found that arthritis (not better specified) was the most common post-COVID-19 manifestation of rheumatological interest, with a prevalence of 37%, even higher than arthralgia or fatigue. Knee (72%), ankle (67%) and wrist (50%) were the most commonly affected joints [61].

### 3.3. Fibromyalgia in Post-COVID-19 Patients

Two studies evaluated the prevalence of fibromyalgia in post-COVID-19 patients. The results of an Italian web-based cross-sectional survey including 616 patients showed that 31% fulfilled the classification criteria for fibromyalgia after an average of 6 months from the diagnosis of COVID-19, whose strongest predictors were obesity and male gender [68]. Using a similar online survey methodology in Sweden, based on self-reported data collected after a mean of 47 weeks from infection, 40% of the participants fulfilled the 2016 criteria for fibromyalgia and 22% reported to be heathy before COVID-19 [32].

### 3.4. Rheumatoid Factor and Anti-Citrullinated Protein Antibodies in Post-COVID-19 Patients

Data about RF and ACPA in the post-COVID-19 period were found in seven articles. To determine the seroprevalence of ACPA after COVID-19, Derksen et al. tested 61 patients visiting their post-COVID-19 outpatient clinic. With the exception of two patients previously diagnosed with ACPA-positive RA, none of the patients tested positive for ACPA [9]. Similarly, in the case series from Ursini et al., the authors concluded that autoantibodies were usually absent. None of the 23 patients with post-COVID-19 inflammatory arthritis presented RF or ACPA positivity [67]. Comparing the prevalence of RF and ACPA in a sample of 33 subjects from a Colombian post-COVID-19 clinic, Acosta-Ampudia et al. noticed that the autoantibody positivity did not change from acute disease to post-COVID-19 assessment at a median time of 266 days and that the frequency of these autoantibodies was not different compared with a control group of healthy individuals [25]. In a cohort of 201 patients with prior COVID-19, Schultheiss et al. described a prevalence of autoantibody positivity close to 20% for RF (data derived from plots – numbers not available) [60]. Comparably, Xu et al. collected sera from 129 COVID-19 patients admitted to hospital [74] and, after excluding patients with a history of RA, RF was detected in 20% of cases. Dynamic changes were further investigated in five patients. Tracking for several months the levels of RF, the authors suggested that RF antibodies peak in the later phase of the disease and last for a long time [74]. Moreover, Lingel et al. also analyzed serum levels of RF and ACPA in specimens from convalescent patients after SARS-CoV-2 infection. The authors found that there was no difference between post-COVID-19 patients and healthy donors or those with acute disease regarding levels of RF, while ACPA were elevated specifically in convalescents when compared to unexposed donors and remained elevated after 8 months [52]. Finally, assessing the rheumatologic manifestations of 100 patients 6 months after the infection and excluding patients with previous autoimmune or rheumatic diseases, Taha et al. found a prevalence of 19% for RF and of 39% for ACPA in Egypt [61]. 

### 3.5. Other Musculoskeletal Inflammatory Conditions of Rheumatological Interest

Ardakani et al. described 5 patients developing septic arthritis with concomitant avascular necrosis of the femoral head, on average 42 days after the infection [30], while performing 18F fluorodeoxyglucose (FDG) positron emission tomography (PET) on 13 post-COVID-19 patients, Kiatkittikul et al. showed radiotracer uptake in the skeletal muscle of 8 patients (62%), compatible with myositis [50].

## 4. Discussion

**Figure 2:**
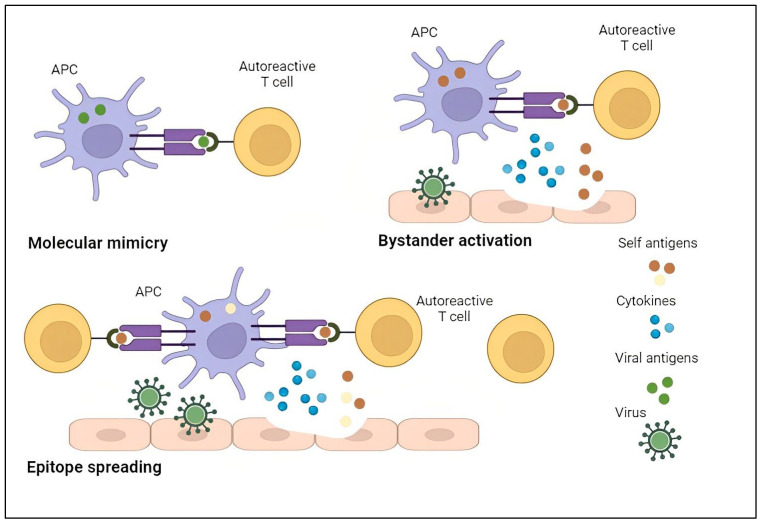
Molecular mimicry is characterized by the presence of epitopes on SARS-CoV-2 spike glycoprotein that cross-react with host antigens leading to the activation of cellular and humoral autoreactivity, due to the similarity to self-epitopes. Bystander activation implies the activation of a non-specific and over-reactive antiviral immune response. This phenomenon leads to the liberation of self-antigens from the damaged tissue and the release of cytokines from the antigen presenting cells (APCs). Self-antigen is then taken up by APCs and presented to T cells. This way, APCs activate autoreactive T cells, exacerbating the tissue destruction. A persistent viral infection can lead to the phenomenon of epitope spreading. In fact, the perpetuation of tissue damage can promote the release of new self-antigens that may activate more autoreactive T cells when taken up by APCs [83].

We performed a systematic review with the aim of shedding light on post-acute COVID-19 musculoskeletal manifestations of rheumatological interest. Most of the included literature reported data about prevalence of joint pain in post-COVID-19 patients. The results were widely heterogeneous, ranging from 2% to 65% with a time frame after acute infection varying from 4 weeks to 12 months, therefore precluding the possibility to perform a quantitative synthesis of the data. Regarding inflammatory arthritis, the available literature described different clinical phenotypes. A symmetrical polyarthritis involving wrists and hands has been reported, with an RA-like pattern reminiscent of other prototypical viral arthritis, while in other patients a different pattern has been observed, with monoarthritis or oligoarthritis of large joints, more similar to reactive arthritis, or PMR-like clinical presentations. Since treatment with non-steroidal anti-inflammatory drugs (NSAIDs) and local steroids has been used for post-COVID-19 arthralgia, a potential explanation of the pain origin could be the activation of peripheral nociceptors, suggesting a nociceptive phenotype [78,79]. Nevertheless, it has also been hypothesized that the damage to connective tissue caused by SARS-CoV-2 leads to widespread pain with nociplastic features, in particular in patients with joint hypermobility [78,79]. Although the precise underlying mechanisms remain to be fully elucidated, growing evidence suggests the association between COVID-19 and the development of autoimmune conditions [17,79]. The role of pathogens in the onset of autoimmune diseases has been demonstrated for different viruses, such as influenza, Ebola, Zika and Chikungunya [80]. Molecular mimicry, bystander killing, epitope spreading, clearance deficiency and viral persistence have all been proposed as factors that increase the risk of autoreactivity and reduce self-tolerance, potentially contributing to the autoimmune phenomena observed after viral infections [81,82,83]. A prototypical example of molecular mimicry underlying virus-induced autoimmunity is the association between Epstein–Barr virus and a variety of autoimmune diseases, such as RA and systemic lupus erythematosus (SLE) [80,84]. Bystander activation is believed to be the responsible mechanism for the association between Hepatitis C virus and Sjögren’s syndrome [80,85]. Additionally, the formation of neutrophilic extracellular traps (NETosis) in COVID-19 can act as a trigger for the development of humoral autoimmunity, especially against intra-nuclear antigens leading to the formation of anti-nuclear antibodies [86]. Finally, in severe COVID-19, the hyper-inflammatory state triggered by exaggerated immune response and activation of multiple inflammatory pathways may lead to the generation of autoimmunity [18]. The main mechanisms that have been postulated to be implied in the self-tolerance disruption induced by viruses are represented in Figure 2.

Moreover, we found studies focusing on fibromyalgia and it should be noticed that, although the available literature is still limited, the proportion of post-COVID-19 patients fulfilling the classification criteria for fibromyalgia is high, ranging from 31% to 40%. Regarding the prevalence of RF and ACPA, largely inconsistent results were found, precluding the possibility to draw conclusions. Interestingly, besides arthralgia or inflammatory rheumatic diseases, a few cases of avascular necrosis of the femoral head or of transient osteoporosis of the hip have been described, suggesting potential vascular implications caused by the endothelial dysfunction induced by SARS-CoV-2 [30,87,88]. The early recognition of patients at higher risk of developing post-COVID-19 musculoskeletal diseases could lead to a better treatment of these conditions. One of the suggested responsible factors is pre-existing musculoskeletal pain. Although patients with rheumatic diseases have demonstrated a strongly resilient attitude during COVID-19 pandemic [89,90], these individuals are at higher risk of exacerbation of the pre-existing pain after COVID-19, but also of developing new onset COVID-19-related pain. Moreover, the presence of myalgia during the acute phase of SARS-CoV-2 infection has also been suggested to be related to a higher risk of developing post-COVID-19 musculoskeletal diseases [91]. The approaches that have been used to contain COVID-related rheumatic musculoskeletal diseases mostly consisted of repurposed pre-existing immunomodulatory drugs used in autoimmune diseases, including corticosteroids, intravenous immunoglobulins and biologic agents [92]. In particular, some studies reporting COVID-19-related acute arthritis proposed a variety of treatments, including corticosteroids, NSAIDs and baricitinib [18]. Other biologic therapies that have been used in critically ill COVID-19 patients include anakinra, an IL-1 receptor antagonist, and tocilizumab, a monoclonal antibody that inhibits the IL-6 receptor, which have both been proven to be beneficial in macrophage activation syndrome (MAS) [80,93]. Nevertheless, none of these treatments has reached sufficiently robust evidence to be introduced in routine practice [86]. An interesting point to consider is the possible effect of anti-SARS-CoV-2 drugs on the development of musculoskeletal and autoimmune conditions [86]. The temporary use of immunosuppressant drugs and their late withdrawal has indeed been related to the paradoxical development of autoimmunity due to the inappropriate immune reconstitution [94]. Additionally, some of the first antiviral drugs used against COVID-19, such as favipiravir and lopinavir-ritonavir combination, may have represented a trigger for gout manifestations [80]. On the other hand, studies have also hypothesized that the use of hydroxychloroquine (HCQ) in the early phase of the pandemic may have prevented or weakened inflammatory joint manifestations [95].

The main findings of our review can be summarized as follows: (1) joint pain is arguably one of the most prevalent complaints in post-COVID-19 patients, with potential long-term implications on quality of life and functional disability; (2) post-COVID-19 inflammatory arthritis and PMR might represent a new entity of musculoskeletal conditions temporally related to SARS-CoV-2 infection, whose disease course and response to treatment need to be determined in prospective studies; (3) SARS-CoV-2 infection might induce loss of tolerance and autoantibody formation triggering RF and ACPA responses, but their pathogenetic role in the risk of developing rheumatic diseases is unclear, constituting an emerging challenge for the rheumatologist.

Several limitations of our review must be considered. The follow-up after acute infection was heterogeneous, but also the definition of post-COVID varied, ranging from onset of first COVID-19 symptoms to first positive nasopharyngeal swab, end of quarantine, negative nasopharyngeal swab, hospital admission or hospital discharge. Furthermore, the vaccination status of these patients was not always reported. Information about COVID-19 vaccination might be relevant when assessing the development of rheumatic musculoskeletal conditions because, for instance, cases of new-onset inflammatory arthritis and PMR after COVID-19 vaccine administration have been described [96,97,98,99]. Even though no relevant changes in the prevalence of long-COVID symptoms between vaccinated and non-vaccinated hospitalized COVID-19 survivors has been observed [100], the topic is still controversial and other studies suggest that a reduction of risk of long-COVID manifestations could be attributed to vaccines [101]. Finally, the heterogeneity of the different populations considered in the studies represented a major limitation to the evaluation of comorbidities and medication use, which are strongly associated with the severity of the disease but could also be able to mitigate the symptoms of covid-related manifestations, thus acting as confounding factors [86,102].

## 5. Conclusions

In conclusion, notwithstanding the abovementioned limitations, our systematic review suggests that manifestations of potential rheumatological interest such as arthralgia, but also established musculoskeletal rheumatic diseases, are frequently reported in post-COVID-19 patients, both early during the convalescent period or later in the recovery course. However, large epidemiological studies are needed to elucidate the association between SARS-CoV-2 exposure and the development of rheumatic musculoskeletal diseases, quantify the true incidence, and explore the pathophysiological mechanism linking SARS-CoV-2 infection to the development of rheumatic diseases. 

## Figures and Tables

**Table 1:** Characteristics of the studies included in the review.

First Author; Year	Study Design	Country	Rheumatic Disease	Time between COVID-19 and RMD	Number of Post-COVID-19 Patients	Percentage of Patients with Arthralgia or RMD or Main Findings about Autoantibodies
Acosta-Ampudia; 2022 [25]	Prospective observational study	Colombia	Arthralgia and autontibodies (RF, ACPA)	Median of 288 days	33	Frequency of RF and ACPA did not differ from healthy subjects.
Akova; 2022 [26]	Cross-sectional observational study	Turkey	Arthralgia	12 months	151	46%
Al-Aly; 2021 [27]	Prospective observational study (electronic health records)	USA	Arthralgia and inflammatory arthritis	>1 month	73,435	N/R
Aly; 2021 [28]	Retrospective cross-sectional observational study	Egypt	Arthralgia	>1 month	115	21%
Anaya; 2021 [29]	Case series	Colombia	Arthralgia	Median of 219 days	100	65%
Ardakani; 2022 [30]	Case series	Iran	Septic arthritis	Mean of 42 days	5	100%
Bakılan; 2021 [31]	Retrospective cross-sectional observational study	Turkey	Arthralgia	>4 weeks	280	36%
Bileviciute-Ljungar; 2022 [32]	Cross-sectional observational study	Sweden	Fibromyalgia	>12 weeks	100	40%
Buonsenso; 2022 [33]	Cross-sectional observational study	UK/USA/other countries	Arthralgia	>1 month	510 children (in 41% COVID-19 was suspected, not confirmed)	61%
Carfì; 2020 [34]	Cross-sectional observational study	Italy	Arthralgia	60 days	143	27%
Carvalho-Schneider; 2021 [35]	Prospective observational study	France	Arthralgia	1 month and 2 months	150 at 1 month and 130 at 2 months	10% at 1 month and 16% at 2 months
Chudzik; 2022 [36]	Prospective observational study	Poland	Arthralgia	>4 weeks	218	2%
Chudzik; 2022 [37]	Retrospective observational study	Poland	Arthralgia	3 months	2,218	4%
Cui; 2022 [38]	Prospective observational study	China	Arthralgia	12 months	1,296	12%
Derksen; 2021 [9]	Case series	Netherlands	Inflammatory arthritis and autoantibodies (ACPA)	5 weeks for determination of ACPA, mean of 7 weeks for onset of arthritis	61 for determination of ACPA; patients across various Dutch rheumatology clinics for RA	0 new ACPA;5 RA
Galal; 2021 [39]	Cross-sectional observational study	Egypt	Arthralgia	Mean of 176 days	430	57%
Gamal; 2022 [40]	Prospective observational study	Egypt	Arthralgia	1.5 months	170	19%
Ghosn; 2021 [41]	Prospective observational study	France	Arthralgia	6 months	1,137	Around 16% at 3 months and 18% at 6 months
Gulzar; 2022 [42]	Cross-sectional observational study	Pakistan	Arthralgia and inflammatory arthritis	6 months	367	25% for arthralgia and 1% for arthritis
Heesakkers; 2022 [43]	Prospective observational study	Netherlands	Arthralgia	12 months	243	26%
Huang C.; 2021 [44]	Ambidirectional observational study	China	Arthralgia	Median of 186 days	1,733	9%
Huang L.; 2021 [45]	Ambidirectional observational study	China	Arthralgia	>6 months	1,225 at 6 months, 1,272 at 12 months	11% at 6 months and 12% at 12 months
Irisson-Mora; 2022 [46]	Cross-sectional observational study	Mexico	Arthralgia	6 months	280	12%
Karaarslan; 2021 [47]	Prospective observational study	Turkey	Arthralgia	1 month	300	22%
Karaarslan; 2022 [48]	Prospective observational study	Turkey	Arthralgia	3 months and 6 months	291 at 3 months and 285 at 6 months	39% at 3 months and 19% at 6 months
Kenny; 2022 [49]	Prospective observational study	Ireland	Arthralgia	>4 weeks	233	23%
Kiatkittikul; 2022 [50]	Retrospective observational study	Thailand	Myositis	Median of 32 days	13	62%
Kim; 2022 [51]	Prospective observational study	Korea	Arthralgia	12 months	170	12%
Lingel; 2021 [52]	Prospective observational study	Germany	Autoantibodies (RF, ACPA)	8 months	68	ACPA were elevated in post-COVID-19 patients compared to unexposed donors and remained elevated in convalescents even after 8 months post infection.
Lombardo; 2021 [53]	Prospective observational study	Italy	Arthralgia	12 months	303	48%
Maestre-Muniz; 2021 [54]	Cross-sectional observational study	Spain	Inflammatory arthritis	≥12 weeks	543	2%
Martone; 2022 [55]	Cross-sectional observational study	Italy	Arthralgia	Mean of 88 days	541 (20% sarcopenic)	36% of non-sarcopenic patients and 24% of sarcopenic patients
Mukarram; 2021 [56]	Case series	Pakistan	Inflammatory arthritis	Mean of 8 weeks	5	100%
Muñoz-Corona; 2022 [57]	Ambidirectional observational study	Mexico	Arthralgia	3 months	141	47%
Petersen; 2021 [58]	Prospective observational study	Denmark	Arthralgia	Mean of 93 days	180	Around 11%
Sarda; 2022 [59]	Cross-sectional observational study	India	Arthralgia	>4 weeks	251	4%
Schultheiss; 2022 [60]	Cross-sectional observational study	Germany	Autoantibodies (RF)	>4 weeks	201	Around 20%
Taha; 2021 [61]	Cross-sectional observational study	Egypt	Inflammatory arthritis, autoantibodies (RF, ACPA)	6 months	100	37% for arthritis; 19% for RF; 39% for ACPA
Tiwari; 2021 [62]	Cross-sectional observational study	Nepal	Arthralgia	2 months	132	6%
Tleyjeh; 2022 [63]	Cross-sectional observational study	Saudi Arabia	Arthralgia	>4 weeks	5,946	31%
Tosato; 2021 [64]	Cross-sectional observational study	Italy	Arthralgia	Mean of 77 days	165	22%
Tuzun; 2022 [65]	Cross-sectional observational study	Turkey	Arthralgia	>4 weeks	113	40%
Uniyal; 2022 [66]	Cross-sectional observational study	India	Arthralgia	>6 weeks	360	21% at 6 weeks—3 months, 29% over 3 months
Ursini; 2021 [67]	Case series	Italy	Inflammatory arthritis and autoantibodies (FR, ACPA)	Mean of 33 days	23	100%
Ursini; 2021 [68]	Cross-sectional observational study	Italy	Fibromyalgia	Mean of 6 months	616	31%
Vaira; 2022 [69]	Cross-sectional observational study	Italy	Arthralgia	>6 Months	431	18%
Vogler; 2022 [70]	Case series	Germany/Italy	Inflammatory arthritis	>4weeks	10	10 (6 without underlying RMD, 4 with underlying RMD)
Wang; 2021 [71]	Cross-sectional observational study (electronic health records)	USA	Arthralgia	>50 days	26,117	21%
Whittaker; 2021 [72]	Population based study	UK	Arthralgia	>4 weeks	456,002	3%
Wong-Chew; 2022 [73]	Prospective observational study	Mexico	Arthralgia	>30 days	4,670	11%
Xu; 2021 [74]	Case series	China	Autoantibodies (RF)	Several months	129	20%
Yaksi; 2022 [75]	Retrospective observational study	Turkey	Arthralgia	4 months	133	25%
Zayet; 2021 [76]	Retrospective observational study	France	Arthralgia	Mean of 289 days	127	24%
Zuschlag; 2022 [77]	Retrospective observational study	Germany	Arthralgia	12 months	162	7%

Legend: ACPA: anti-citrullinated protein antibodies; RF: rheumatoid factor; RMD: rheumatic musculoskeletal disease.

**Table 2:** Newcastle-Ottawa scale for quality appraisal of non-randomized observational studies.

First Author; Year	Representativeness of the Exposed Cohort	Selection of the Non-Exposed Cohort	Ascertainment of Exposure	Demonstration That Outcome of Interest Was Not Present at Study Entry	Comparability of Cohorts on the Basis of the Design or Analysis	Assessment of Outcome	Was Follow-Up Long Enough for Outcomes to Occur	Adequacy of Follow-Up of Cohorts	Total Quality Score	Quality
Acosta-Ampudia; 2022 [25]	1	0	1	0	1	1	1	1	6	fair
Akova; 2022 [26]	0	1	1	0	1	1	0	0	5	fair
Al-Aly; 2021 [27]	1	1	1	1	2	1	1	1	9	good
Aly; 2021 [28]	1	0	1	0	1	1	1	0	5	fair
Bakılan; 2021 [31]	1	0	1	0	0	1	0	0	3	poor
Bileviciute-Ljungar; 2022 [32]	1	1	1	0	1	1	1	0	6	fair
Buonsenso; 2022 [33]	1	1	0	1	0	0	1	1	5	fair
Carfì; 2020 [34]	1	1	1	1	2	1	1	1	9	good
Carvalho-Schneider; 2021 [35]	1	1	1	0	1	1	1	1	7	good
Chudzik; 2022 [36]	1	1	0	0	1	1	1	0	5	fair
Chudzik; 2022 [37]	1	0	0	1	1	1	1	0	5	fair
Cui; 2022 [38]	1	1	1	1	0	0	1	0	6	fair
Galal; 2021 [39]	1	0	0	1	0	1	0	0	3	poor
Gamal; 2022 [40]	1	0	1	1	0	1	1	0	5	fair
Ghosn; 2021 [41]	1	1	1	1	1	1	1	1	8	good
Gulzar; 2022 [42]	1	0	1	0	1	0	0	0	3	poor
Heesakkers; 2022 [43]	1	1	1	1	2	1	1	1	9	good
Huang C.; 2021 [44]	1	1	1	1	2	1	1	1	9	good
Huang, L.; 2021 [45]	1	1	1	1	2	1	1	1	9	good
Irisson-Mora; 2022 [46]	1	0	1	0	0	1	0	1	4	fair
Karaarslan; 2021 [47]	1	0	1	0	0	1	0	1	4	fair
Karaarslan; 2022 [48]	1	1	1	0	1	1	0	0	5	fair
Kenny; 2022 [49]	0	1	1	1	2	0	0	1	6	fair
Kiatkittikul; 2022 [50]	1	1	1	0	0	0	0	1	4	fair
Kim; 2022 [51]	1	1	1	1	1	1	1	0	7	good
Lingel; 2021 [52]	1	1	1	1	2	1	1	1	9	good
Lombardo; 2021 [53]	1	0	1	1	1	1	1	1	7	good
Maestre-Muniz; 2021 [54]	1	1	1	1	1	1	1	1	8	good
Martone; 2022 [55]	1	0	1	1	2	1	1	0	7	good
Muñoz-Corona; 2022 [57]	1	0	1	0	0	1	1	0	4	fair
Petersen; 2021 [58]	1	1	0	1	1	0	1	1	6	fair
Sarda; 2022 [59]	1	0	1	0	0	1	1	0	4	fair
Schultheiss; 2022 [60]	1	1	1	1	1	1	1	0	7	good
Taha; 2021 [61]	0	1	1	0	1	1	1	1	6	fair
Tiwari; 2021 [62]	1	0	0	0	0	1	0	0	3	poor
Tleyjeh; 2022 [63]	1	1	1	1	1	1	0	1	7	good
Tosato; 2021 [64]	1	1	1	0	1	1	1	1	7	good
Tuzun; 2022 [65]	1	0	1	1	0	1	0	0	4	fair
Uniyal; 2022 [66]	1	1	0	0	0	0	1	0	3	poor
Ursini; 2021 [68]	1	1	1	1	2	1	1	1	9	good
Vaira; 2022 [69]	0	1	1	1	1	1	1	0	6	fair
Wang; 2021 [71]	1	0	1	1	1	0	1	0	6	fair
Whittaker; 2021 [72]	1	1	1	1	2	1	1	1	9	good
Wong-Chew; 2022 [73]	1	0	1	1	0	1	0	0	4	fair
Yaksi; 2022 [75]	1	1	1	0	0	0	1	1	5	fair
Zayet; 2021 [76]	1	1	1	1	1	1	1	1	8	good
Zuschlag; 2022 [77]	1	1	1	0	0	1	1	1	6	fair

## Data Availability

No new data were created or analyzed in this study. Data sharing is not applicable to this article.
